# Experimental study on dynamic characteristics of tailings under different consolidation conditions

**DOI:** 10.1038/s41598-023-42532-0

**Published:** 2024-01-06

**Authors:** Changbo Du, Xinqi Jiang, Laigui Wang, Fu Yi, Ben Niu

**Affiliations:** 1 College of Civil Engineering, Liaoning Technical University, Fuxin, 123000 China; 2 School of Mechanics and Engineering, Liaoning Technical University, Fuxin, 123000 Liaoning China; 3 Beijing Jingneng Geological Engineering Co.,Ltd, Beijing, 102300 China

**Keywords:** Natural hazards, Engineering

## Abstract

The dynamic stability of tailing ponds depend largely on the dynamic characteristics of tailings sand. To explore the dynamic characteristics of tailings sand under different consolidation conditions, consolidated undrained triaxial tests under different dry densities, consolidation ratios and containing pressures, the dynamic shear stress, liquefaction stress ratio, dynamic strength index, dynamic pore water pressure, dynamic modulus, and damping ratio of tailings sand under different consolidation conditions were analyzed. The dynamic shear stress linearly changed with the number of failure vibrations. The liquefaction stress ratio increases with an increase in consolidation ratio, conforming to the quadratic polynomial of the origin. With an increase in failure vibration times, the dynamic internal friction angle decreases gradually. Under different failure vibration times, the dynamic internal friction angle increases with an increase in consolidation ratio and dry density. An exponential function model of dynamic pore pressure growth suitable for equal pressure and bias consolidation conditions is proposed, and the fitting effect is favorable. The dynamic shear modulus ratio decreases with an increase in dynamic shear strain; the damping ratio increases with an increase in dynamic shear strain. The research results can provide a theoretical reference for seismic liquefaction of tailings dams in high-intensity seismic areas.

## Introduction

The statistical analysis of 3500 tailing dams worldwide^1^ shows that dam failure is approximately 10 times more likely to occur for tailing dams than in water-retaining dams, causing a major disaster to the lives and property of people downstream and to the surrounding area. Earthquakes are the second leading cause of tailing dam disasters^2^. Earthquake disasters lead to liquefaction of settling ponds, resulting in the breach of the settling dam and threatening the safety of local residents. Therefore, it is very important to analyze the dynamic stability of the tailing pond construction area. The dynamic stability of the tailing dam largely depends on the dynamic characteristics of the tailings sand in dams^3^, so it is necessary to study the dynamic characteristics of the tailings sand.

Tailings sand is usually in a saturated loose state, the particles are finer and the specific gravity is greater, which is due to the influence of particle composition, mineral composition, and other factors. They appear to be stable but are actually very sensitive to disturbance. They are susceptible to liquefaction and destructive deformation during earthquakes^4^. At present, the research on the mechanical properties of tailings sand is mainly focused on static properties, while the research results on the dynamic properties are not very numerous. Chen et al.^5^ took an iron ore tailings silt as a research object. Through the dynamic triaxial test, it was found that initial liquefaction occurs when the dynamic strain amplitude reaches 5%. The dynamic shear stress increases with the increase of confining pressure. However, the dynamic shear stress is not sensitive to the confining pressure. Jame et al.^6^ reached the same conclusion by a uniaxial test of non-plastic gold tailings sand. Liu et al.^7^ investigated the dynamic strength characteristics of copper mine tailings silt by a dynamic triaxial test. Zhang et al.^8^ studied the effect of fine particle content on the liquefaction characteristics of tailings. It was found that the dynamic strength of tailings initially increases and then decreases with increasing fine particle content. Naeini et al.^9^ and Geremew et al.^10^ studied the effect of fine particle content and mineral composition on the liquefaction characteristics of tailings through laboratory tests and numerical analysis. Tan et al.^11^ used a dynamic triaxial test to investigate the dynamic characteristics of tailings silt under different consolidation ratios and found that the saturated tailings silt was completely liquefied under isobaric consolidation, whereas the saturated tail silt did not liquefy during bias consolidation. Yu et al.^12^ determined the fitting relationship between the dynamic strength, dynamic pore pressure, and failure vibration ratio of tailings sand under different consolidation ratios and consolidation stress conditions using a dynamic triaxial test. Jame et al.^13^ investigated the liquefaction characteristics and methods for predicting the liquefaction of gold mine tailings under earthquake conditions through cyclic single-shear experiments. Chu et al.^14^ investigated the effect of fine particle content on the dynamic characteristics of tailings sand by a dynamic triaxial test. It is found that at constant vibration times, the dynamic shear stress ratio of tailings sand first increases and then decreases with increasing fine particle content. Jin et al.^15^ investigated the liquefaction characteristics of tailings sand and the dynamic response characteristics of tailings dam under earthquake conditions by a shaking table model test. Liu et al.^16^ conducted a dynamic triaxial test on zinc ore tailings in a closed system and investigated the dynamic pore pressure and cyclic dynamic characteristics of tailings. Payan et al.^17,18^ conducted several experiments on silt to study its dynamic characteristics. A new expression and a model of dynamic characteristics of silt were proposed.

The soil properties of tailings sand differ from those of general sands, especially the particle composition and the physicochemical effect of membrane water in the pores. Its dynamic characteristics are obviously different from those of sand, and the dynamic strength and dynamic pore pressure growth mode are also different. The dynamic characteristics of tailings sand under different consolidation conditions are rarely being studied. Under the action of dynamic load, the dynamic characteristics and laws of different tailings are often different. In this study, consolidated undrained dynamic triaxial tests are performed under different dry densities, consolidation ratios, and confining pressures. The dynamic shear stress, liquefaction stress ratio, dynamic strength index, dynamic pore water pressure, and dynamic modulus damping ratio of whole-tailings under different consolidation conditions are analyzed. The dynamic characteristics, dynamic pore pressure development law, and the dynamic shear modulus fitting relationship of tailings sand under equal pressure and bias pressure are determined, to provide a scientific basis for seismic stability analysis of tailings dam design.

## Test equipment and samples

### Sample preparation and saturation

**Figure 1:**
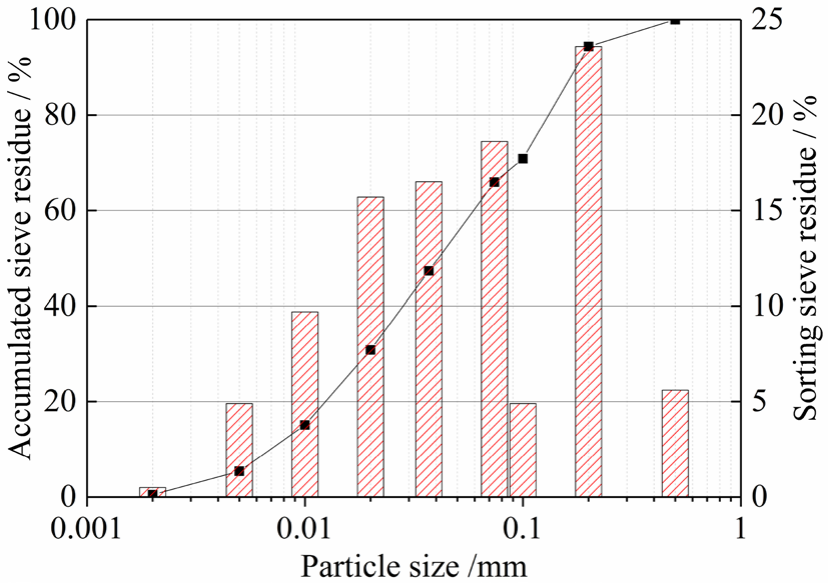
Particle grading curve of tailings sand.

The total tailings sand used in the test was obtained from a tailings pond in Fujian Province. The density was 1.83 g/cm^3^, moisture content was 3.75%, Poisson ratio *v* was 0.42, and the elastic modulus *E* was 1.6 × 105 Pa. The physical property index of the tailings sand was as follows: effective grain size d_10_ = 0.10 mm, median particle size d_30_ = 0.19 mm, and constrained grain size d_60_ = 0.30 mm. According to the calculation, the uneven coefficient of the tailing *C*_u_ was 3 (< 5), and the coefficient of curvature *C*_c_ was 1.2 (between 1 and 3); this shows that the tailings were poorly graded, and the particle grading curve is shown in Fig. 1.

Place the tailings sand in a moisturizing cylinder, let it infiltrate for 24 h, and then measure its moisture content. According to the moisture content and the required dry density of the sample, it is divided into five layers, which are compacted and shaped in the sampler. To avoid stratification between the layers, the contact surfaces of each layer are etched, and after hitting the last layer, the sample is removed and weighed. The parallel error of each sample is less than 0.02 g/cm^3^. The sample was formed into a Φ 39.1 × 80 mm cylindrical sample with a certain moisture content. The compacted sample was then loaded into a saturator. The saturator was inserted into the vacuum pump equipment for pumping as it approaches atmospheric pressure and then pump for 2 h. Then, distilled water is slowly injected and the sample is left to stand for more than 96 h after suction saturation. After the sample is saturated by the above method, the degree of saturation is above 0.95, and then it is consolidated according to the required stress state.

### Test conditions and methods

**Figure 2:**
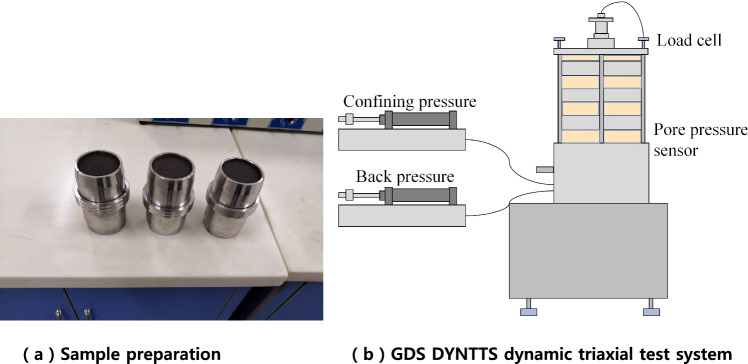
Sample preparation and schematic of experimental setup.

A GDS DYNTTS dynamic triaxial test system was used for this test (shown in Fig. 2). The standard used in this test is ' Soil Test Procedure'(SL237-1999) issued by the Ministry of Water Resources of the People's Republic of China. According to the standard test, the vibration frequency is 1 Hz^19^. If the change in consolidation displacement within 1 h is not more than 0.1 cm^3^, the consolidation time is considered to be completed at approximately 12 h. If the consolidation pressure is equal (*K*_c_ = 1.0), the full amplitude strain is 5%, and if the consolidation pressure is not equal (*K*_c_ > 1.0), the comprehensive strain is 5%.

The samples were consolidated under confining pressure *σ*_3_ = 100, 150, 200 kPa. Each group of dynamic loads consists of 5 to 6 levels, and each level of dynamic load vibration is 10 times. The dynamic stress and strain at each level of the dynamic load are recorded. The specific test method is consistent with another article^20^ written by the author.

### Test scheme

Dynamic tests were performed on the whole tailings with consolidation stress ratios *K*_c_ of 1.0, 1.5, and 2.0 under various stress conditions. The corresponding confining pressures are 100, 150, and 200 kPa, respectively. Three different dynamic stresses are selected for each stress state. The specific test scheme is listed in Table 1.

**Table 1:** Dynamic test scheme for tailings.

Test scheme		*K*_c_	*ρ*_d_/(g/cm^3^)	*σ*_3_/kPa	Vibration times
Isotropic consolidation	a	1.0	1.58	100, 150, 200	10, 20, 30
	b		1.68		
	c		1.79		
Anisotropic consolidation	d	1.5	1.58		
	e		1.68		
	f		1.79		
	g	2.0	1.58		
	h		1.68		
	i		1.79		

## Results and discussion

### Variation law of dynamic shear stress

During the test, the dynamic shear stress is calculated according to the following equation:

1 $$\tau_{{\text{d}}} = \frac{1}{2}\sigma_{{\text{d}}}$$

where $\tau_{{\text{d}}}$ is dynamic shear stress, $\sigma_{{\text{d}}}$ is axial dynamic stress amplitude, and the corresponding dynamic stress value when the strain reaches 5%.

**Figure 3:**
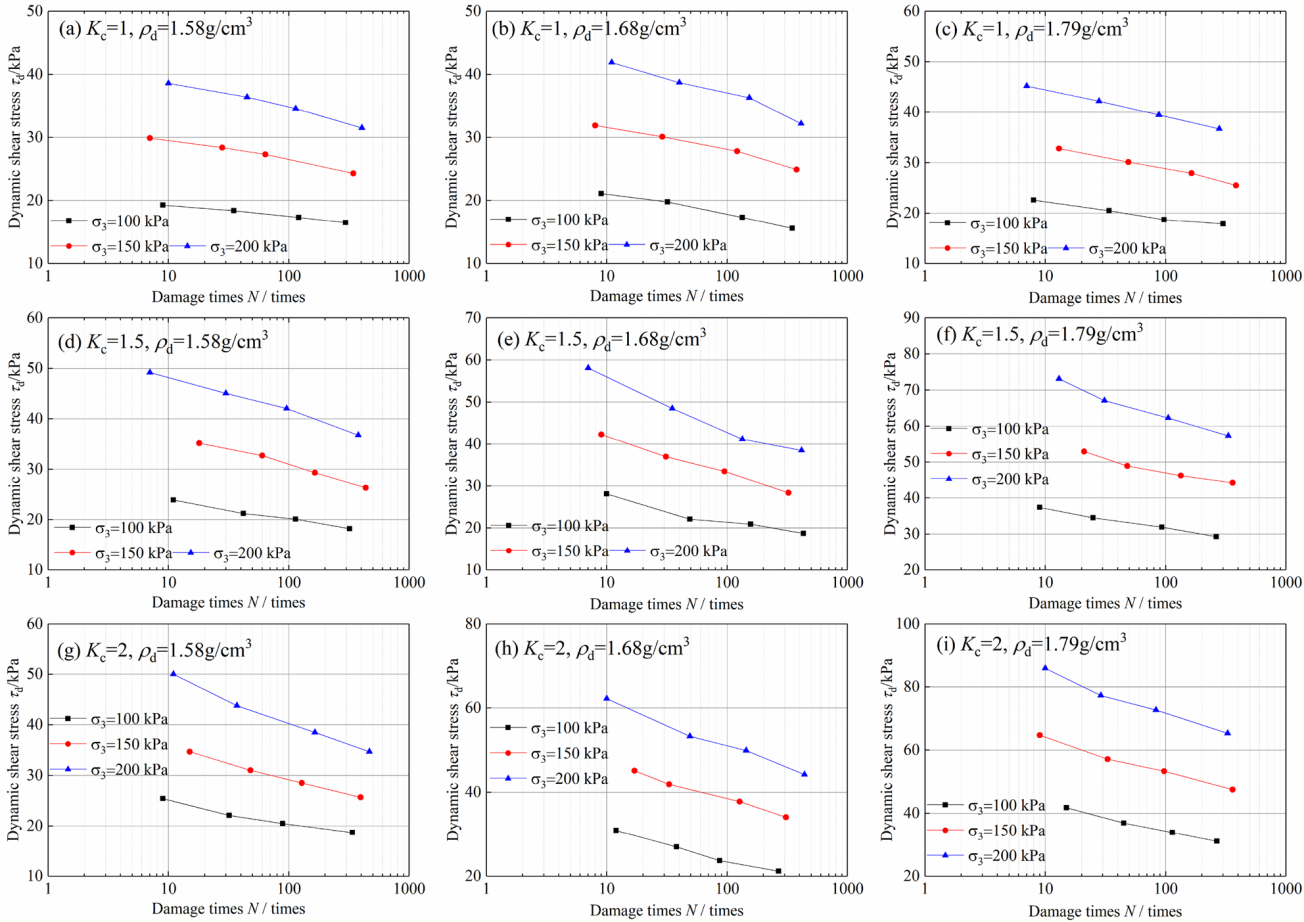
Variation relationship between the dynamic shear stress of tailings and failure vibration times.

Figure 3 shows the relationship between the dynamic shear stress and failure vibration number of tailings sand. The test parameters are linearly fitted as follows:

2 $$\tau_{{\text{d}}} = a_{{1}} + b_{{1}} \ln N$$

where *a*_1_ and *b*_1_ are the experimental constants.

The fitting results of model parameters are shown in Fig. 3, and the model fit parameters are shown in Table 2. It can be seen from Fig. 3 that the dynamic shear stress of tailings sand has a linear relationship with the failure vibration times. The fitting degree of the test data is very high, and the correlation coefficient reaches more than 95%.

**Table 2:** Test constants of the fitting formula between the dynamic shear stress and failure vibration times.

*K*_c_	*ρ*_d_/(g/cm^3^)	σ_3_ = 100 kPa			σ_3_ = 150 kPa			σ_3_ = 200 kPa		
		*a*_1_	*b*_1_	*R*^*2*^	*a*_1_	*b*_1_	*R*^*2*^	*a*_1_	*b*_1_	*R*^*2*^
1	1.58	21.15	− 1.86	0.992	32.99	− 3.23	0.974	43.31	− 4.39	0.975
	1.68	24.72	− 3.52	0.98	35.89	− 4.11	0.972	48.22	− 5.88	0.96
	1.79	25.24	− 3.08	0.967	38.29	− 4.85	0.990	49.78	− 5.32	0.999
1.5	1.58	27.7	− 3.79	0.984	43.75	− 6.53	0.984	55.44	− 7.05	0.987
	1.68	32.9	− 5.56	0.914	50.51	− 8.78	0.995	66.79	− 11.33	0.964
	1.79	42.37	− 5.39	0.993	61.27	− 6.88	0.942	81.42	− 10.90	0.979
2	1.58	28.96	− 4.2	0.957	41.89	− 6.3	0.994	59.08	− 9.25	0.989
	1.68	38.57	− 7.33	0.981	55.19	− 8.43	0.990	72.47	− 10.72	0.986
	1.79	51.44	− 8.49	0.989	74.12	− 10.53	0.985	98.11	− 13.23	0.977

### Variation of the liquefaction stress ratio

The liquefaction standard of this test is that when the strain reaches 5%, the liquefaction stress ratio is equal to the ratio of confining pressure to axial dynamic stress amplitude:

3 $$\frac{{\tau_{{\text{d}}} }}{{\sigma_{{3}} }} = \frac{{\sigma_{{\text{d}}} }}{{{2}\sigma_{{3}} }}$$

where $\tau_{{\text{d}}} {/}\sigma_{{3}}$ is the liquefaction stress ratio and $\sigma_{{3}}$ is confining pressure.

The liquefaction stress ratio of tailings sand under different conditions is calculated according to Eq. (3), as shown in Table 3. From the table, it can be seen that the confining pressure has little effect on the liquefaction stress ratio. This is because the particles of the tailings sand are poorly rounded, and have a large surface area. Compared to natural sand, the contact area between adjacent skeleton particles is larger^21^. During consolidation, the relative position of the particles is gradually adjusted. Finally, a ' force chain ' of tight consolidation of the structure is formed, which can transfer the consolidation stress and dynamic stress more uniformly to each skeleton particle. Therefore, even if the confining pressure is different, the same liquefaction stress ratio results in the same dynamic strength. This is consistent with the research results of Yin^21^.

**Table 3:** Liquefaction stress ratio of tailings.

σ_3_/kPa	*ρ*_d_/ (g/cm^3^)	*K*_c_	Stress ratio of liquefaction						Fitting formula	*R*^2^
			10 weeks	20 weeks	30 weeks			mean value		
100/150/200	1.58	1	0.1930	0.1900		0.1880	0.1903		$\tau_{{\text{d}}} {/}\sigma_{{3}} = K_{{\text{c}}} \left( { - 0.0722K_{{\text{c}}} + 0.2613} \right)$	0.999
		1.5	0.2400	0.2330		0.2250	0.2327			
		2	0.2510	0.2370		0.2230	0.2370			
	1.68	1	0.2100	0.2050		0.2000	0.2050		$\tau_{{\text{d}}} {/}\sigma_{{3}} = K_{{\text{c}}} \left( { - 0.0{563}K_{{\text{c}}} + 0.2613} \right)$	0.999
		1.5	0.2810	0.2590		0.2470	0.2623			
		2	0.3110	0.2960		0.2800	0.2957			
	1.79	1	0.2200	0.2150		0.2100	0.2150		$\tau_{{\text{d}}} {/}\sigma_{{3}} = K_{{\text{c}}} \left( { - 0.0{257}K_{{\text{c}}} + 0.2613} \right)$	0.973
		1.5	0.3700	0.3530		0.3370	0.3533			
		2	0.4300	0.4070		0.3850	0.4073			

**Figure 4:**
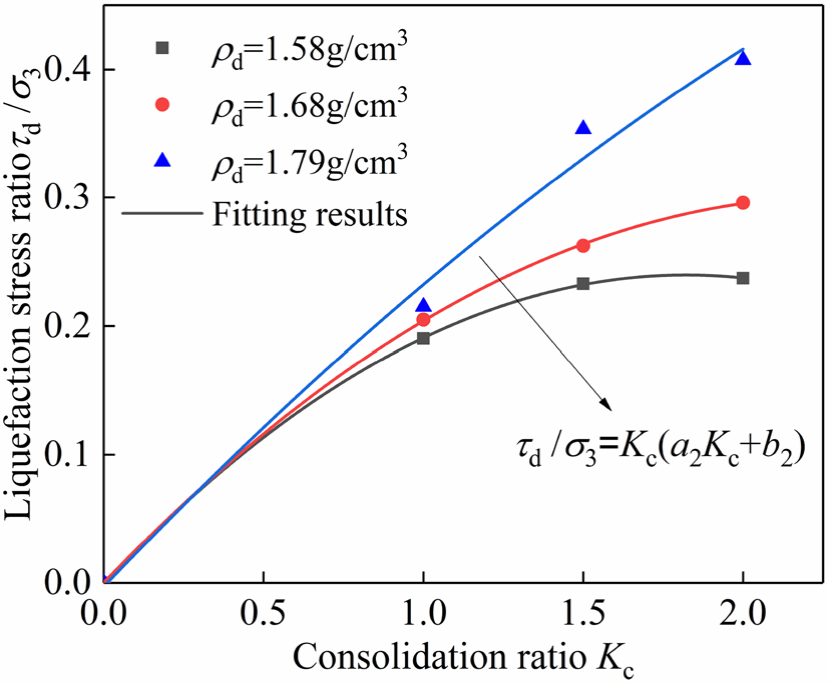
Variation relationship between the liquefaction stress ratio and consolidation ratio of tailings under different dry density conditions.

The relationship between the liquefaction stress ratio and consolidation ratio of tailings sand under different dry density conditions is analyzed as shown in Fig. 4. From the figure, it can be seen that the effect of dry density on the liquefaction stress ratio is not very clear. The larger the consolidation ratio, the stronger the influence of dry density on the liquefaction stress ratio. The effect of the consolidation ratio on the liquefaction stress ratio is clear, the liquefaction stress ratio increases with the increase of the consolidation ratio. Since the test curve has a consolidation ratio of 0, the liquefaction stress ratio is also 0. Quadratic polynomial fitting of the curve through the origin is as follows:

4 $$\tau_{{\text{d}}} {/}\sigma_{{3}} = K_{{\text{c}}} \left( {a_{{2}} K_{{\text{c}}} + b_{{2}} } \right)$$

where *a*_2_ and *b*_2_ are the fitting parameters.

The fitting results are shown in Fig. 4, which shows the relationship between the liquefaction stress ratio and consolidation ratio corresponding to a quadratic polynomial through the origin. The fitting effect is good, and the correlation coefficient is above 99%. When the dry density is 1.58 g/cm^3^, the liquefaction stress ratio is $\tau_{{\text{d}}} {/}\sigma_{{3}} = K_{{\text{c}}} \left( { - 0.0722K_{{\text{c}}} + 0.2613} \right)$, and the dry density is 1.68 g/cm^3^, the liquefaction stress ratio is $\tau_{{\text{d}}} {/}\sigma_{{3}} = K_{{\text{c}}} \left( { - 0.0{563}K_{{\text{c}}} + 0.2613} \right)$. Further analysis shows that as the dry density increases, the peak values of the liquefaction stress ratio and the corresponding consolidation ratio also increase, the polynomial fitting parameter *a*_2_ also increases, and *b*_2_ remains unchanged at 0.2613.

### Dynamic strength index changes

The dynamic strength of soil refers to the dynamic stress required for the soil to produce a specified strain under a given number of stress cycles. The Mohr–Coulomb theory of shear strength is still applicable to vibration. The dynamic shear stress *τ*_d_, which corresponds to 10, 20, and 30 times the failure vibration times under three different confining pressures is plotted on the curve of dynamic shear stress and failure vibration times with the same consolidation ratio. Taking the dynamic shear stress *τ*_d_ as the ordinate, the principal stress σ as the abscissa, the $\left( {\sigma_{1c} + \sigma_{3c} } \right){/2}$ as the center of the circle, and the $\left( {\sigma_{1c} - \sigma_{3c} } \right){/2}$ as the radius, the envelope of the total stress shear strength is drawn, the dynamic internal friction angle *φ*_d_ and dynamic cohesion *c*_d_ at different damage vibration times are obtained as follows^22^:

5 $$\tau_{{\text{d}}} = \sigma_{{\text{d}}} \cdot \tan \phi_{{\text{d}}} + c_{{\text{d}}}$$

The test results show the dynamic strength index of tailings sand (dynamic cohesion and dynamic internal friction angle) under different conditions in Table 4. From the table, it can be seen that the dynamic cohesion is basically not affected by the three factors mentioned above, because the tailings sand is a non-sticky granular material, and its value is 0. The change in dynamic internal friction angle is significantly affected by the consolidation ratio and is less affected by the vibration times and dry density. The dynamic internal friction angle decreases gradually with an increase in failure vibration time. Under different failure vibration times, the dynamic internal friction angle increases with an increase in consolidation ratio and dry density, which is consistent with the results reflected by the dynamic strength curve (see Fig. 3).

**Table 4:** Dynamic strength indexes of tailings under different conditions.

*K*_c_	*ρ*_d_/ (g/cm^3)^	10 cycles		20 cycles		30 cycles		Mean value	
		*c*_d_/ kPa	*φ*_d_/ kPa	*c*_d_/ kPa	*φ*_d_/ kPa	*c*_d_/ kPa	*φ*_d_/ kPa	*c*_d_/ kPa	*φ*_d_/ kPa
1	1.58	0	9.3	0	9.2	0	9.1	0	9.2
	1.68	0	10	0	9.8	0	9.6	0	9.8
	1.79	0	10.4	0	10.2	0	10	0	10.2
1.5	1.58	0	19.2	0	19	0	18.8	0	19
	1.68	0	20.3	0	19.7	0	19.4	0	19.8
	1.79	0	22.5	0	22.1	0	21.7	0	22.1
2	1.58	0	25.4	0	25.1	0	24.8	0	25.1
	1.68	0	26.6	0	26.3	0	26	0	26.3
	1.79	0	28.8	0	28.4	0	28	0	28.4

**Figure 5:**
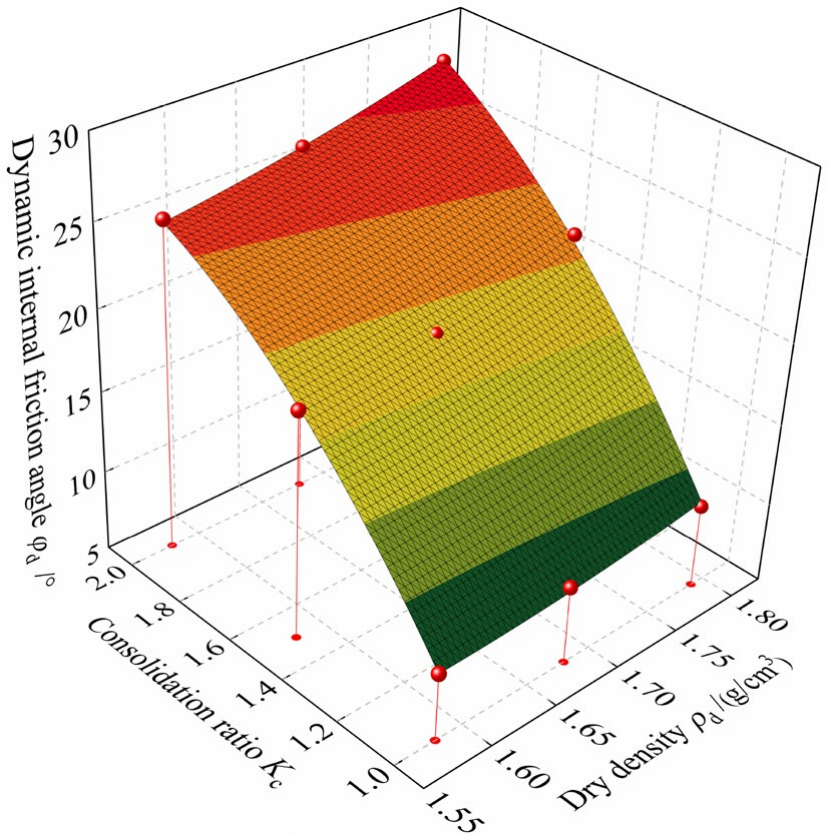
Variation relationship between the dynamic internal friction angle of tailings and consolidation ratio and dry density.

This study focuses on the influence of consolidation ratio and dry density on the dynamic characteristics of tailings. According to Table 4, the three-dimensional surface fitting relationship between the dynamic internal friction angle of tailings sand and the consolidation ratio and dry density without considering the vibration times is shown in Fig. 5. It can be seen from the figure that the three-dimensional surface fitting formula is $\phi_{{\text{d}}} { = 63}{\text{.1 + 23}}{.0}K_{{\text{c}}} { - 99}{\text{.1}}\rho_{{\text{d}}} { - 8}{\text{.5}}K_{{\text{c}}}^{{2}} { + 28}{\text{.0}}\rho_{{\text{d}}}^{{2}} { + 11}{\text{.0}}K_{{\text{c}}} \rho_{{\text{d}}}$, and the correlation coefficient is 99.7%.

### Dynamic pore water pressure change

#### Development law of dynamic pore pressure

**Figure 6:**
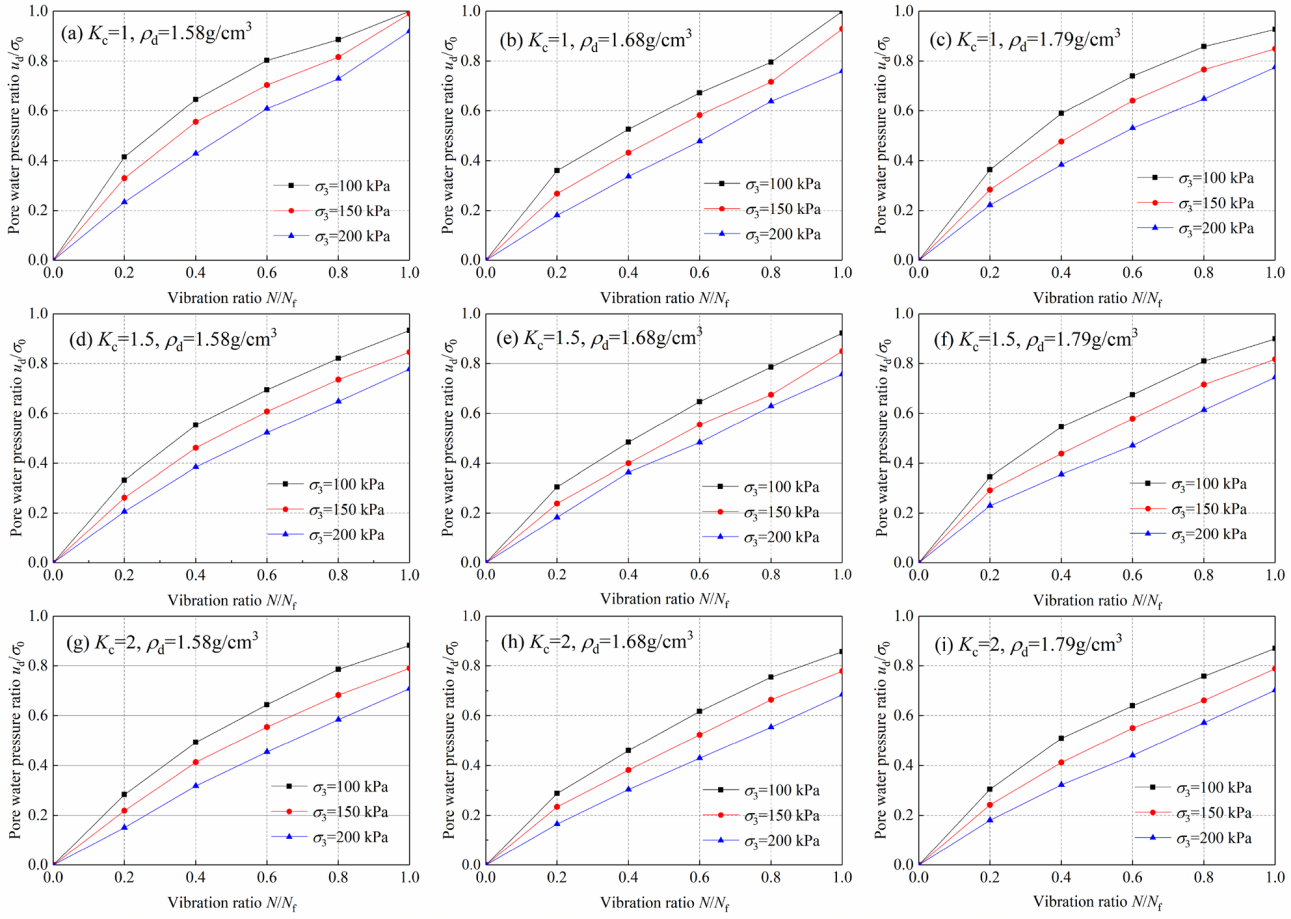
Relationship between the dynamic pore water pressure ratio *u*_d_/*σ*_0_ and vibration ratio *N*/*N*_f_ of tailings.

The dynamic pore pressure ratio *u*_d_/*σ*_0_ is used to represent the change in dynamic pore pressure, and the ratio of vibration times to failure times *N*/*N*_f_ is used to represent the ratio of vibration times (*u*_d_ is the dynamic pore water pressure, MPa; *σ*_0_ is the initial effective consolidation stress, $\sigma_{{0}} = (\sigma_{{1}} + \sigma_{{3}} )/2$; *N* is the vibration time; *N*_f_ is the vibration number at the time of liquefaction damage). The consolidated undrained dynamic triaxial test was analyzed, and the relationship curves between the dynamic pore pressure ratio *u*_d_/σ_0_ and the vibration ratio *N*/*N*_f_ of the tailings sand under equal pressure and eccentric pressure were obtained, as shown in Fig. 6. It can be seen from the figure that under the action of dynamic load, the dynamic pore pressure ratio *u*_d_/*σ*_0_ gradually increases with an increase in the vibration ratio* N*/*N*_f_, and the increase is accompanied by an increase in the deformation of the tailings sand test. In the isobaric consolidation (*K*_c_ = 1), the final stage of the increase in the dynamic pore pressure ratio is abrupt. In the case of bias consolidation (*K*_c_ ≥ 1), the development trend of the dynamic pore pressure ratio gradually moves downward, and the mutation in the final stage disappears. The larger the consolidation ratio, the lower the critical dynamic pore pressure. For the same tailings material, the influence of confining pressure and dry density on the dynamic pore pressure growth curve is not obvious, while the consolidation ratio has a significant influence on the dynamic pore pressure growth curve.

Figure 6a–c shows the relationship between the dynamic pore pressure ratio *u*_d_/*σ*_3_ and the vibration ratio *N*/*N*_f_ in the case of isobaric consolidation (*K*_c_ = 1). In the case of isobaric consolidation, a relatively small dynamic load can easily lead to liquefaction failure of saturated sand. Because $u_{{\text{d}}} = \sigma_{{0}} = \left( {\sigma_{{1}} + \sigma_{{3}} } \right){/2} = \sigma_{{3}}$ the dynamic pore pressure is close to the initial effective consolidation stress when the liquefaction occurs, the dynamic pore pressure ratio *u*_d_/*σ*_3_ is approximately equal to 1. Under this consolidation condition, the dynamic pore pressure ratio *u*_d_/*σ*_3_ and the vibration ratio *N*/*N*_f_ basically change from 0 to 1, and the regularity is obvious. At present, the most studied dynamic pore pressure model of saturated sand is isobaric consolidation. Figure 6d–h shows the relationship between the dynamic pore pressure ratio *u*_d_/*σ*_3_ and the vibration ratio *N*/*N*_f_ of the tailings sand in the case of bias consolidation (*K*_c_ = 1.5 and 2). In the case of bias consolidation (*K*_c_ = *σ*_1_/*σ*_3_ > 1), a larger dynamic load is required to cause liquefaction damage in the saturated sand due to the large axial stress. When compared with isobaric consolidation, the rising law of dynamic pore pressure is basically the same as that for isobaric consolidation. However, in the case of isobaric consolidation, owing to $u_{{\text{d}}} = \sigma_{{0}} = \left( {\sigma_{{1}} + \sigma_{{3}} } \right){/2} = \left( {K_{{\text{c}}} + 1} \right)\sigma_{{3}} /2$,$u_{{\text{d}}} = \sigma_{{3}} = {2}\sigma_{{0}} {/}\left( {K_{c} + 1} \right)$_,_ the dynamic pore pressure ratio changes between 0 and ${2/}\left( {K_{c} + 1} \right)$ during liquefaction failure, so the rising law of dynamic pore pressure is slower than that of isobaric consolidation.

### Dynamic pore pressure growth model

Seed et al.^23^ performed undrained dynamic triaxial tests on saturated sand under isotropic consolidation. Based on the test results, the dynamic pore pressure stress model for general saturated sand under isobaric consolidation is proposed, and its applicability is widely accepted:

6 $$\frac{{u_{d} }}{{\sigma_{{0}} }} = \frac{2}{\pi }\arcsin \left( {\frac{N}{{N_{{\text{f}}} }}} \right)^{1/\theta }$$

where *θ* is an experimental constant, usually 0.7.

The above equation was proposed for general saturated sand and is obviously not suitable for tailings sand. Later, further studying tailings sand, the dynamic pore pressure model for tailings sand was also proposed^24,25^. However, this model is only valid for isobaric consolidation and is no longer applicable to eccentric consolidation. A dynamic pore pressure growth exponential function model is proposed, which is suitable for both isobaric consolidation and anisotropic consolidation, as follows:

7 $$\frac{{u_{d} }}{{\sigma_{{0}} }} = a_{{3}} \left( {1 - \exp ( - b_{{3}} \frac{N}{{N_{{\text{f}}} }})} \right)$$

where *a*_3_ and *b*_3_ are experimental constants.

The experimental curves of dynamic pore pressure ratio *u*_d_/*σ*_0_ and vibration ratio *N*/*N*_f_ of tailings sand under different conditions were fitted with the exponential function model of dynamic pore pressure growth. The fitting results of model parameters are shown in Table 5. It can be seen from the table that the model has a high agreement of fitting to the test data, and the correlation coefficient reaches more than 99%.

**Table 5:** Dynamic pore pressure growth index model fitting test constant.

Fitting parameters		*ρ*_d_ = 1.58 g/cm^3^			*ρ*_d_ = 1.68 g/cm^3^			*ρ*_d_ = 1.79 g/cm^3^		
		σ_3_ = 100 kPa	σ_3_ = 150 kPa	σ_3_ = 200 kPa	σ_3_ = 100 kPa	σ_3_ = 150 kPa	σ_3_ = 200 kPa	σ_3_ = 100 kPa	σ_3_ = 150 kPa	σ_3_ = 200 kPa
*K*_c_ = 1	*a*_3_	1.0904	1.2842	2.0726	1.3326	2.0085	2.6246	1.066	1.1295	1.3456
	*b*_3_	2.2596	1.3709	0.5719	1.2553	0.5919	0.343	2.0233	1.4007	0.8427
	*R*^2^	0.997	0.993	0.997	0.980	0.989	0.999	0.999	0.999	0.999
*K*_c_ = 1.5	*a*_3_	1.1452	1.2105	1.4756	1.3212	1.7289	1.8702	1.0625	1.1052	1. 4436
	*b*_3_	1.6199	1.1842	0.7381	1.1585	0.6528	0.5149	1.7983	1.3063	0.7045
	*R*^2^	0.998	0.999	0.999	0.996	0.995	0.998	0.997	0.994	0.991
*K*_c_ = 2	*a*_3_	1.2112	1.2902	2.5671	1.1944	1.3713	2.3127	1.0852	1.1865	1.8883
	*b*_3_	1.2975	0.9449	0.3237	1.2466	0.829	0.3474	1.5502	1.0578	0.4579
	*R*^2^	0.999	0.999	0.999	0.998	0.997	0.999	0.997	0.998	0.998

### Variation of dynamic modulus and damping ratio

#### Maximum dynamic elastic modulus and maximum dynamic shear modulus

Because the relationship between stress and strain of tailings sand is obviously nonlinear, the relationship between strain amplitude and stress under periodic load can be approximated as a hyperbolic curve, as follows:

8 $$\sigma_{{\text{d}}} = \frac{{\varepsilon_{{\text{d}}} }}{{a_{{4}} + b_{{4}} \cdot \varepsilon_{{\text{d}}} }}$$

Rewrite this equation as:

9 $$E_{{\text{d}}} = \frac{{\sigma_{{\text{d}}} }}{{\varepsilon_{{\text{d}}} }} = \frac{1}{{a_{{4}} + b_{{4}} \cdot \varepsilon_{{\text{d}}} }}$$

Let Eq. (9) take the limit at both ends:

10 $$E_{{d\max }} = \mathop {\lim }\limits_{{\varepsilon _{d} \to 0}} \frac{1}{{a_{4} + b_{4} \cdot \varepsilon _{d} }} = \frac{1}{{a_{4} }}$$

where $E_{{\text{d}}}$ is the dynamic elastic modulus; $\varepsilon_{{\text{d}}}$ is axial strain amplitude; $\sigma_{{\text{d}}}$ is the dynamic stress corresponding to $\varepsilon_{{\text{d}}}$; *a*_4_ and *b*_4_ are experimental parameters.

Draw the ${1/}E_{{\text{d}}}$ ~ $\varepsilon_{{\text{d}}}$ relationship curve, take the intercept *a*_4_ on the ordinate, and its reciprocal is the maximum dynamic elastic modulus $E_{{{\text{d}}\max }}$. According to the principle of material mechanics, the shear elastic modulus *G*_d_ is:

11 $$G_{{\text{d}}} = \frac{{E_{{\text{d}}} }}{{2\left( {1 + \mu } \right)}}$$

where $\mu$ is the Poisson ratio of tailings sand, the strain of saturated tailings sand is zero $\mu 0.5$.

Based on the test results, the maximum dynamic elastic modulus *E*_dmax_ and the maximum dynamic shear modulus *G*_dmax_ of tailings sand under different conditions are calculated. The specific data are listed in Table 6. The maximum dynamic elastic modulus *E*_dmax_ and the confining pressure are nondimensionalized. The two are in a linear relationship in the double logarithmic coordinates, as follows :

12 $$\frac{{E_{{{\text{dmax}}}} }}{{{\text{Pa}}}} = k\left( {\frac{{\sigma_{3} }}{{{\text{Pa}}}}} \right)^{n}$$

**Table 6:** Maximum dynamic elastic modulus and maximum shear modulus of tailings.

*K*_c_	*ρ*_d_/(g/cm^3^)	σ_3_/kPa	*a*_4_	*E*_dmax_/MPa	*G*_dmax_/MPa	Experimental parameters		*γ*_m_(× 10^–4^)
						*k*	*n*	
1	1.58	100	0.005	202	67	1.8598	0.8994	2.4428
		150	0.0041	241	80			3.5326
		200	0.0028	359	120			3.9368
	1.68	100	0.0049	204	68	1.8873	0.9388	1.9034
		150	0.004	251	84			2.3877
		200	0.0027	373	124			2.5902
	1.79	100	0.0039	256	85	2.5375	0.5745	1.7995
		150	0.0032	316	105			2.2340
		200	0.0026	380	127			2.9761
1.5	1.58	100	0.004	251	84	2.5171	0.5815	1.2552
		150	0.0031	320	107			1.6547
		200	0.0027	376	125			2.5362
	1.68	100	0.0036	276	92	2.8269	0.4673	1.4984
		150	0.0028	355	118			1.7900
		200	0.0026	384	128			2.5706
	1.79	100	0.0036	276	92	2.7163	0.5282	2.3240
		150	0.003	328	109			3.1555
		200	0.0025	396	132			3.7020
2	1.58	100	0.0036	279	93	2.7935	0.5442	3.5543
		150	0.0029	349	116			4.8194
		200	0.0025	407	136			5.9921
	1.68	100	0.0031	324	108	3.2432	0.3604	1.3032
		150	0.0027	376	125			2.1005
		200	0.0024	416	139			2.9945
	1.79	100	0.0029	342	114	3.3011	0.5292	3.4670
		150	0.0026	386	129			4.4443
		200	0.002	488	163			5.2987

Substituting Eq. (11) into Eq. (12), the similar law of maximum dynamic shear modulus *G*_dmax_ is obtained as follows:

13 $$\frac{{G_{{{\text{dmax}}}} }}{Pa} = \frac{k}{{{2(1} + \mu {)}}}(\frac{{\sigma_{3} }}{Pa})^{n}$$

where *P*a is the atmospheric pressure, take 100 kPa; *k* and *n* are experimental parameters, and their values are listed in Table 6. The table shows that under the same conditions, *k* increases with the increase of the consolidation ratio of the dry density, which is positively correlated, *k* > 1.

### Dynamic shear modulus ratio and damping ratio

The characteristic test of the dynamic shear modulus and damping ratio is the basis of the dynamic response analysis. The test curve can reflect the nonlinear and viscous characteristics of the stress–strain relationship of tailings sand under dynamic load. Xie^26^ proposed the curves of dynamic shear modulus ratio and dynamic strain, damping ratio and dynamic strain, and pointed out that the curves are discrete when the dynamic stress is large. The relationship curve between dynamic shear modulus ratio and dynamic strain, namely the Hardin–Drnevich hyperbolic model, is suitable for sand with small strain and particle size, such as tailings sand, as shown below:

14 $$G_{{\text{d}}} /G_{\max } = 1/\left( {1 + \gamma /\gamma_{{\text{m}}} } \right)$$

The relationship between damping ratio and dynamic strain is shown below:

15 $$\lambda = \lambda_{\max } = ({1 - }G_{{\text{d}}} /G_{\max } ) = 1/\left( {1 + \gamma_{{\text{m}}} /\gamma } \right)$$

where *γ* and *γ*_m_ are the dynamic shear strain and reference shear strain, respectively, *λ* and *λ*_max_ are the damping ratio and its maximum value, respectively.

**Figure 7:**
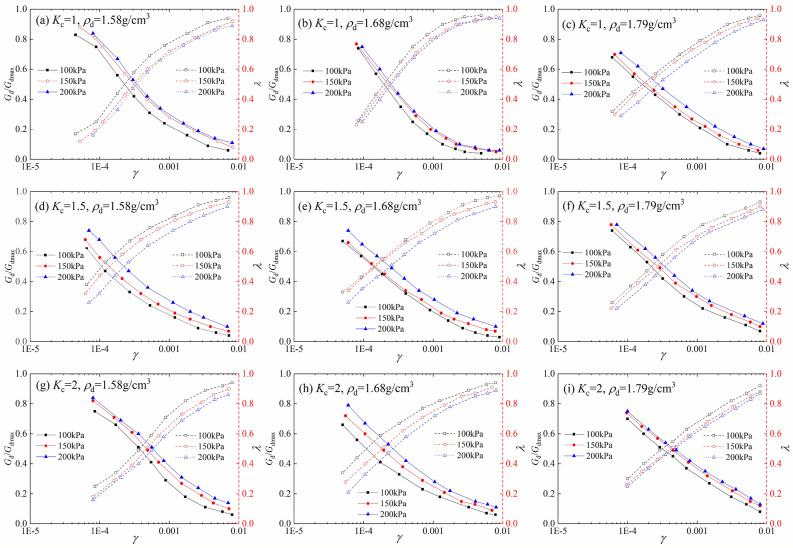
Variation relationship between dynamic shear modulus and dynamic strain of tailings.

From the above analysis, the curves of dynamic shear modulus ratio and damping ratio versus dynamic strain of tailings sand can be obtained, as shown in Fig. 7. The calculated reference shear strains *γ*_m_ are listed in Table 6. As can be seen from the figure, the whole sample is in the elastic stage, when the dynamic strain is less than 10^–4^. The relationship between the dynamic shear modulus ratio and damping ratio under different consolidation stresses does not change significantly. When the dynamic strain is greater than 10^–4^, the dynamic shear modulus ratio decreases under different consolidation stresses, but the larger the consolidation stress, the larger the dynamic shear modulus ratio. The damping ratio of the sample decreases with the increase of the consolidation stress under the same dynamic strain.

**Figure 8:**
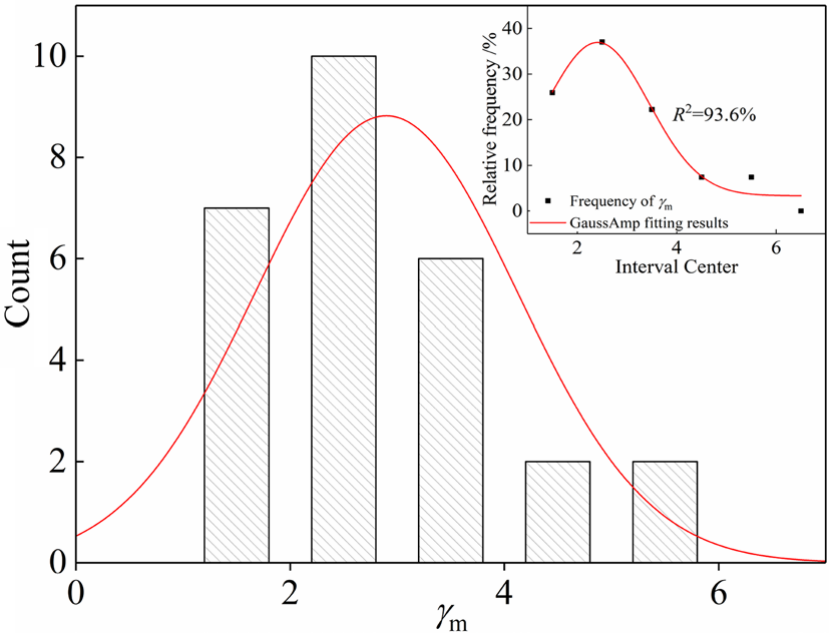
Normal distribution law of reference shear strain.

According to Table 6, the reference shear strain *γ*_m_ was analyzed under different conditions. As can be seen in Fig. 8, the variation of *γ*_m_ under different conditions essentially conforms to the normal distribution. The reference shear strain *γ*_m_ varies from 1.2 to 5.3 *γ*_m_ and increases with increasing confining pressure, consolidation ratio, and dry density.

An exponential function model of pore water pressure growth of tailings sand that is suitable for both isobaric and eccentric consolidation conditions is proposed, and the fitting effect is good.

## Discussion

In this study, as the research object, the whole tailings sand was obtained from a tailings pond in Fujian Province. Dynamic consolidated undrained dynamic triaxial tests under different dry density, consolidation ratio, and confining pressure conditions were used to study the dynamic shear stress, liquefaction stress ratio, dynamic strength index, dynamic pore water pressure, and dynamic modulus damping ratio of tailings sand under different consolidation conditions. The research results provide basic data for the seismic stability analysis and evaluation of the tailings pond as well as valuable insights into the dynamic properties of the tailings and provide a reference for other similar tailings pond projects.

Owing to the differences in particle size, particle morphology, mineral composition, density, structural characteristics, etc. of the tailings, under the action of dynamic load, the dynamic characteristics and laws of different tailings are often different. Therefore, in the seismic analysis of specific tailings dam projects, the specific morphological characteristics of tailings should be discussed in detail and in-depth.

## Conclusion

1. The dynamic shear stress of tailings sand is linearly related to the failure frequency, and the fitting degree of the test data is very high. The slope has a small variation range, and the intercept increases with an increase in consolidation ratio and dry density. The liquefaction stress ratio increases with an increase in consolidation ratio, the two conform to the quadratic polynomial passing through the origin. The change in dynamic internal friction angle is significantly affected by the consolidation ratio and is less affected by the vibration times and dry density. Dynamic cohesion is not affected by these three.
2. The confining pressure and dry density do not distinctly affect the dynamic pore pressure growth curve, while the consolidation ratio significantly affects the dynamic pore pressure growth curve. The rising law of dynamic pore pressure in bias consolidation is slower than that in isobaric consolidation. An exponential functional model of the pore pressure growth of tailings sand is proposed, it is suitable for both isobaric consolidation and bias consolidation conditions and has a good fitting effect.
3. The relationship between dynamic shear modulus and tailings sand decreases with the increase of dynamic shear strain, and the damping ratio increases with the increase of dynamic shear strain, it can be represented by the Hardin–Drnevich hyperbolic model. The relationship between the dynamic shear modulus ratio and damping ratio with dynamic strain is not sensitive to confining pressure and dry density but is obviously affected by the consolidation ratio. The reference shear strain varies between 1.2 and 5.3, and the variation of reference shear strain under different conditions is essentially in accordance with the normal distribution.

## Data Availability

All relevant data are within the paper.

## Abbreviations

*C*_u_: The uneven coefficient of the tailing
[Inline Image Removed]: Poisson ratio
*E*: Elastic modulus
*d*_10_: Effective grain size
*d*_30_: Median particle size
*d*_60_: Constrained grain size
*C*_c_: Coefficient of curvature
*K*_c_: Consolidation stress ratios
τ_d_: Dynamic shear stress
σ_d_: Axial dynamic stress amplitude
ρ_d_: Dry density
τ_d/_σ_3_: The liquefaction stress ratio
σ_3_: Confining pressure
*u*_d_/*σ*_0_: The dynamic pore pressure ratio
*u*_d_: The dynamic pore water pressure
*σ*_0_: The initial effective consolidation stress
*E*_*d*_: The dynamic elastic modulus
_*Ɛ*__d_: Axial strain amplitude
_*E*__d max_: The maximum dynamic elastic modulus
_*G*__d_: The shear elastic modulus
_*a2\b2*_: Fitting parameters
φ_d_: Dynamic internal friction angle
*c*_d_: Dynamic cohesion
θ,* a*_*3*_, *b*_*3*_: Test constant
*K*,* n*: Experimental parameters
*γ*, *γ*_m_: Dynamic shear strain and reference shear strain
λ, λ_max_: Damping ratio and its maximum value
*h*: Hour
